# Early-phase semi-quantitative analysis versus full time-course quantitative modeling of ultrafast dynamic contrast-enhanced MRI for breast cancer diagnosis, molecular subtyping, and treatment response prediction

**DOI:** 10.1186/s13244-025-02166-4

**Published:** 2025-12-17

**Authors:** Ying Cao, Xueqin Gong, Yao Huang, Huifang Chen, Jie Fang, Lu Wang, Lan Li, Sun Tang, Ting Yin, Xiaoxia Wang, Jiuquan Zhang

**Affiliations:** 1 School of Medicine, Chongqing University, Chongqing, China; 2 Department of Radiology, Chongqing University Cancer Hospital, Chongqing Key Laboratory for Intelligent Oncology in Breast Cancer (iCQBC), Chongqing, China; 3 MR Collaborations, Siemens Healthineers Ltd., Chengdu, China

**Keywords:** Breast neoplasms, Magnetic resonance imaging, Differential diagnosis, Neoadjuvant chemotherapy

## Abstract

**Objectives:**

To evaluate the performance of semi-quantitative analysis versus quantitative pharmacokinetic analysis applied to ultrafast dynamic contrast-enhanced (UF-DCE) MRI in: (1) differentiating benign from malignant breast lesions, (2) molecular subtyping, and (3) predicting pathologic complete response (pCR) following neoadjuvant chemotherapy.

**Materials and methods:**

This prospective noninferiority study enrolled 339 consecutive participants with suspected breast lesions between September 2022 and February 2024. All underwent breast DCE MRI with a temporal resolution of 4.5 s, totaling 100 phases. Using the initial 30 phases (early-phase UF-DCE), five semi-quantitative parameters were calculated: wash-in slope (WIS), time-to-peak, bolus arrival time, peak enhancement intensity (PEI), and initial area under the curve in 60 s. Furthermore, three quantitative parameters: volume transfer constant, rate constant (*k*_*ep*_), and extravascular extracellular space volume, were derived from the 100-phase dataset (full time-course UF-DCE). Diagnostic performance was assessed using areas under the curve (AUC) and the DeLong test, with Pearson analysis evaluating the correlation between semi-quantitative and quantitative parameters.

**Results:**

All semi-quantitative and quantitative parameters showed differences between benign and malignant breast lesions (*p* < 0.001). Semi-quantitative WIS demonstrated noninferior diagnostic performance to quantitative *k*_*ep*_ in differentiating benign from malignant lesions (AUC: 0.93 vs 0.92; ∆AUC = 0.02, *p* = 0.35). However, neither approach effectively distinguished molecular subtypes or predicted pCR (*p* > 0.05). Strong correlations were observed in PEI and *K*^*trans*^ (r = 0.75, *p* < 0.001).

**Conclusion:**

Semi-quantitative analysis of early-phase UF-DCE exhibits noninferior performance to quantitative analysis of full time-course UF-DCE MRI for distinguishing benign from malignant breast lesions. Both analytical approaches showed limited utility in molecular subtyping and pCR prediction.

**Critical relevance statement:**

Early-phase UF-DCE MRI provides a cost-effective alternative to full time-course UF-DCE MRI for differentiating benign and malignant breast lesions, demonstrating noninferior diagnostic performance with reduced scan time and no need for pharmacokinetic modeling.

**Key Points:**

- Systematic comparison of early-phase UF-DCE and full time-course UF-DCE MRI for diagnosis, subtyping, and response prediction in a single breast cancer cohort remains limited.
- Early-phase UF-DCE MRI demonstrated noninferior diagnostic performance to full time-course UF-DCE MRI in differentiating benign and malignant breast lesions.
- Early-phase UF-DCE MRI is a time-efficient alternative to full time-course UF-DCE MRI for clinical implementation.

**Graphical Abstract:**

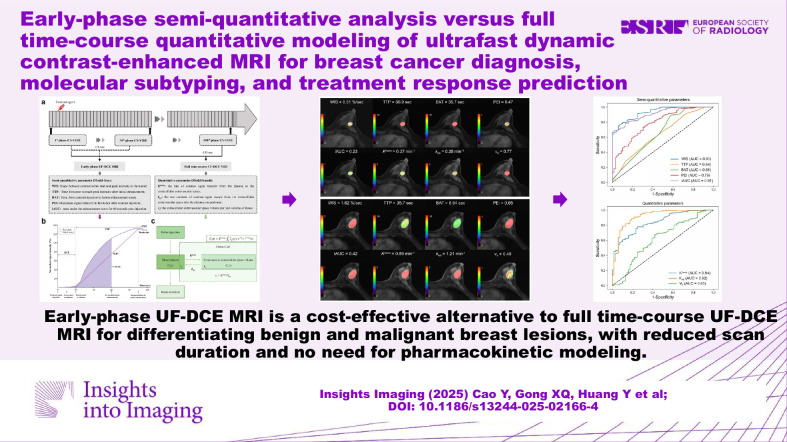

## Introduction

Breast cancer, the most prevalent malignancy among women globally, necessitates precise lesion evaluation for optimal clinical management [1]. The initial diagnostic step involves distinguishing benign from malignant breast lesions, as their management strategies diverge significantly. Benign lesions may only require observation or minimal intervention, whereas malignancies typically demand aggressive treatment, including surgery, chemotherapy, or radiotherapy. Molecular subtyping of breast cancer further refines treatment strategies, providing a basis for personalized treatment and predicting chemotherapeutic responses, thereby optimizing treatment selection [2]. Furthermore, neoadjuvant chemotherapy (NAC) has been increasingly utilized with its advantages of tumor downstaging and increased rates of breast-conserving surgery. Achieving pathologic complete response (pCR) following NAC is a well-established predictor of long-term survival, making accurate pCR prediction crucial for tailoring treatment plans effectively [3].

Dynamic contrast-enhanced (DCE) MRI has emerged as a pivotal tool in breast cancer evaluation due to its high sensitivity and ability to quantify contrast agent kinetics. DCE MRI quantifies vascular permeability and extracellular volume using pharmacokinetic models such as the Tofts model to derive quantitative parameters (e.g., *K*^*trans*^, *k*_*ep*_, *v*_*e*_), which require prolonged data acquisition time (≥ 5 min) to ensure accurate curve fitting [4, 5]. While these quantitative parameters have demonstrated potential in differentiating malignant from benign breast lesions, molecular subtyping of breast cancer, and predicting pCR following NAC [6–8], their widespread clinical adoption is constrained by the need for high temporal resolution (< 15 s/phase) and long scan durations.

Recent advancements in MRI technology have spurred the development of ultrafast DCE MRI (UF-DCE MRI), which offers a short scan time (usually ≤ 2 min) with exceptionally high temporal resolution (3–7 s), capturing early contrast enhancement dynamics within the first 2 min post-injection [9]. This technique derives multiple semi-quantitative parameters that reflect early contrast uptake patterns without pharmacokinetic modeling. These early-phase semi-quantitative parameters may provide a more sensitive assessment of intratumoral perfusion heterogeneity, and emerging studies have reported their potential in differential diagnosis, molecular subtyping, and pCR prediction of breast cancer [10, 11]. Despite these advances, the performance of semi-quantitative analysis based on the early enhancement phase has not yet been comprehensively compared with full time-course quantitative modeling using a single DCE MRI acquisition.

We hypothesize that early-phase semi-quantitative analysis is noninferior to the full time-course quantitative analysis of UF-DCE MRI in breast lesion characterization while offering the advantage of reduced scan duration and no requirement for pharmacokinetic modeling. Therefore, this study aims to comprehensively compare the performance of semi-quantitative parameters versus quantitative parameters obtained from a single UF-DCE acquisition within the same cohort across three key tasks: (1) differentiating malignant from benign breast lesions (task 1), (2) classifying molecular subtypes of breast cancer (task 2), and (3) predicting pCR following NAC (task 3).

## Materials and methods

### Study participants

**Fig. 1:**
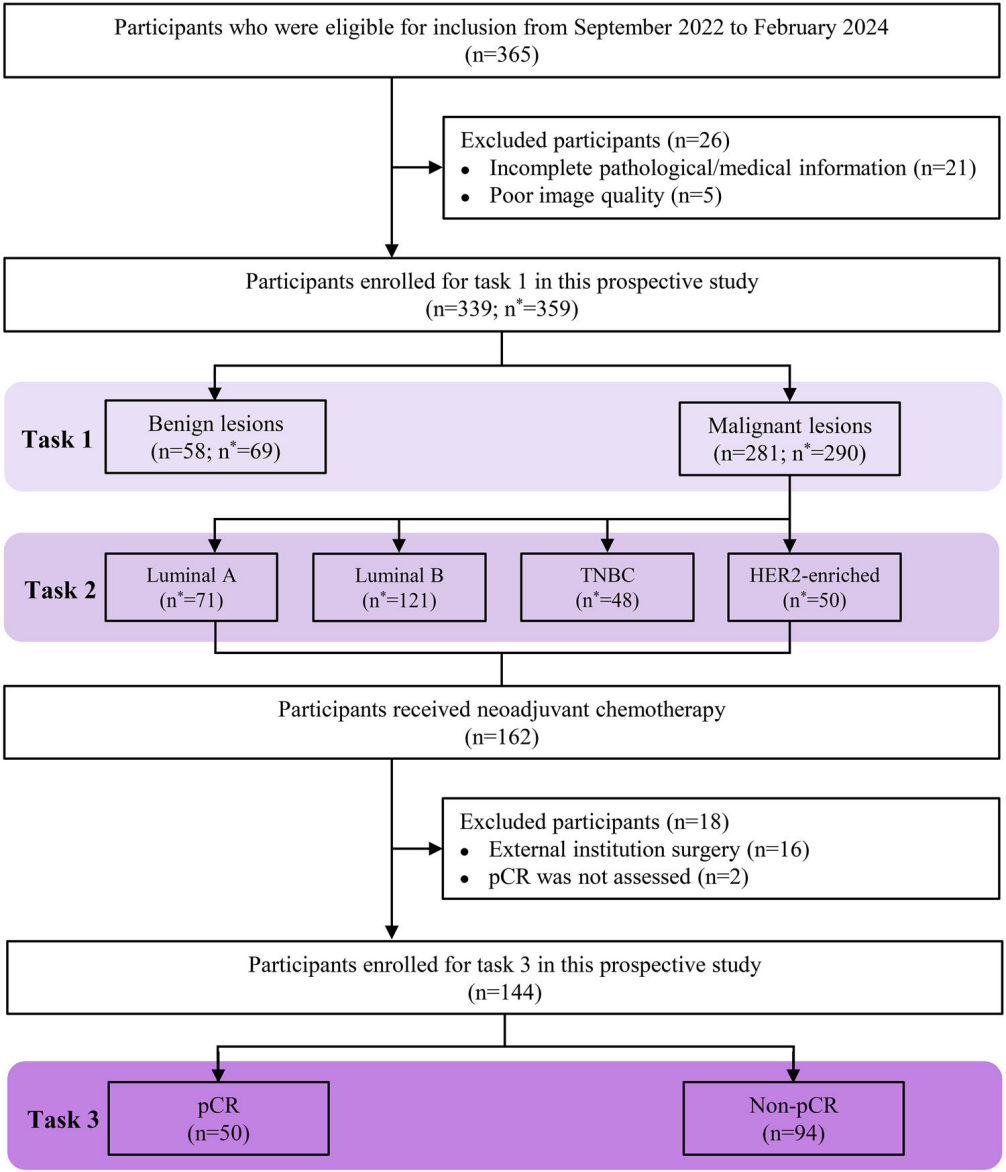
Flow diagram of participant selection. n represents the number of participants, and n* represents the number of lesions

This prospective study was approved by the Institutional Review Board of our hospital (Chongqing University Cancer Hospital (IRB number: CZLS20200215-A)), and all participants provided written informed consent. Between September 2022 and February 2024, consecutive participants meeting clinical indications for breast MRI [12, 13] were enrolled. These indications encompassed: (1) high-risk population screening: Individuals with a family history of breast cancer, mammographically dense parenchyma, previous breast abnormalities, or symptoms warranting biopsy; (2) diagnostic and preoperative evaluation for participants with suspicious findings on mammography and/or ultrasound (Breast Imaging Reporting and Data System (BI-RADS) category ≥ 4), palpable masses, or multifocality assessment. Inclusion criteria were: (1) no prior breast surgery; (2) no previous biopsies or treatment for breast cancer; and (3) histologically confirmed benignancies or malignant diagnosis. Exclusion criteria included: (1) incomplete pathological/medical records (*n* = 21); (2) insufficient image quality (*n* = 5); (3) external institution surgery (*n* = 16); and (4) pCR status was not assessed (*n* = 2). A detailed participant selection flowchart is provided in Fig. 1.

### MRI protocol

MRI scans were performed on a 3-Tesla MRI system (MAGNETOM Prisma, Siemens Healthineers) equipped with a dedicated 16-channel bilateral breast coil. Participants were positioned prone with the head-first orientation. The MRI protocol encompassed pre-contrast T1-weighted imaging, fat-suppressed T2-weighted imaging, diffusion-weighted imaging, and high-temporal resolution DCE sequences. Detailed parameters are listed in Supplementary Table S1.

**Fig. 2:**
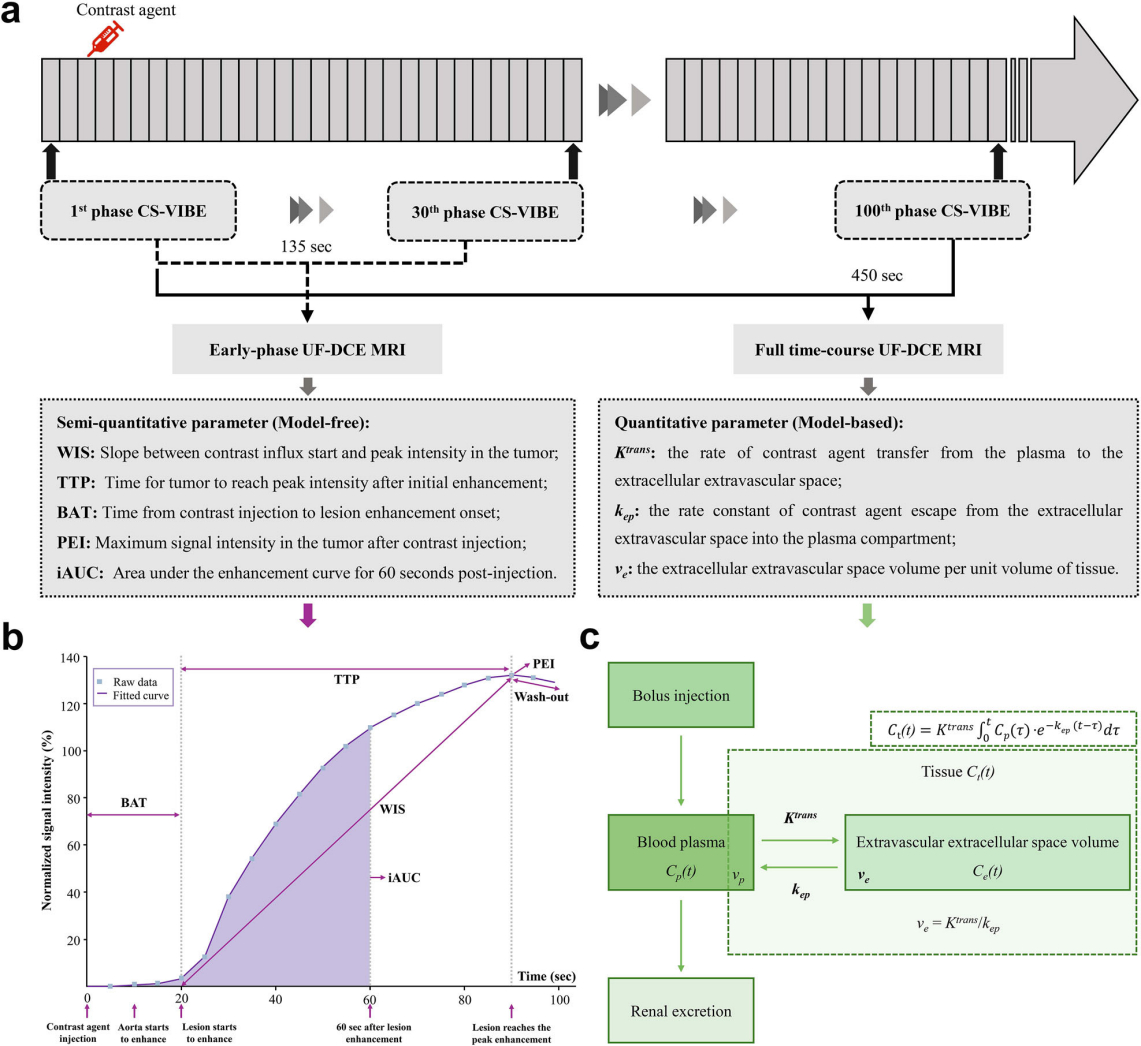
An overview of dynamic contrast-enhanced MRI processing workflow and parameter estimation. **a** Schematic depiction of ultrafast DCE (UF-DCE) MRI acquisition protocols. **b** Representative time-signal intensity curve of early-phase UF-DCE MRI and semi-quantitative parameters. **c** Pharmacokinetic modeling framework applied in full time-course ultrafast dynamic contrast-enhanced MRI quantitative analysis. CS-VIBE, T1-weighted compressed-sensing volume interpolated breath-hold examination; UF-DCE, ultrafast dynamic contrast-enhanced

We implemented a DCE MRI acquisition using compressed sensing accelerated T1-weighted gradient echo acquisition with Dixon water-fat separation. This single continuous protocol maintained high temporal resolution (4.5 s/phase) throughout 100 dynamic phases (total 450 s). From this identical acquisition dataset, the initial 30 phases (representing the first 135 s post-contrast) were extracted as the “early-phase UF-DCE” dataset for semi-quantitative analysis. The entire 100-phase dataset (450 s post-contrast) was utilized for quantitative pharmacokinetic analysis, termed “full time-course UF-DCE” throughout the manuscript. A detailed schema is depicted in Fig. 2a.

Gadobutrol meglumine (Jia Di Xian^®^, Heng Rui) was administered intravenously at a dose of 0.2 mL/kg (0.1 mmol/kg) and a rate of 2.0 mL/s, followed by a 20 mL saline flush at the same rate.

### Image interpretation

Two experienced radiologists (H.F.C. and X.Q.G., with 7 and 5 years of experience in breast MRI, respectively) independently analyzed two datasets (early-phase UF-DCE and full time-course UF-DCE) on a dedicated workstation (Syngovia VB20A, Siemens Healthcare). Both radiologists were blinded to the pathological findings and the results of their own or the other radiologist’s analysis of the alternative sequence. Any delineation discrepancies between radiologists were resolved by consensus.

For the early-phase UF-DCE analysis, the initial 30-phase series was imported, with automatic motion correction applied to align voxels across phases. A region of interest (ROI) was manually defined by the radiologists on the central slice of the tumor’s largest cross-section, with areas of necrosis, cystic changes, or liquefaction excluded. In cases with unilateral multifocal or multicentric lesions, the lesion with the largest diameter was selected; for participants with bilateral disease, the largest lesion from each breast was included for analysis. The defined ROI was then automatically propagated to adjacent slices, forming a spherical volume of interest (VOI). From the segmented VOI, five semi-quantitative parameters were derived: wash-in slope (WIS), time-to-peak (TTP), bolus arrival time (BAT), peak enhancement intensity (PEI), and initial area under the curve in 60 s (iAUC) (Fig. 2b, Appendix S1) [10]. Following the early-phase UF-DCE analysis, the same radiologist consecutively performed the analysis for the full time-course UF-DCE. The methodology mirrored that of the early-phase UF-DCE analysis, but with the inclusion of the entire 100-phase dataset. The same VOI delineation criterion was applied. Quantitative parameters, including *K*^*trans*^, *k*_*ep*_, and *v*_*e*_, were calculated by fitting the Tofts model [14] using a population-averaged arterial input function (AIF) (Fig. 2c, Appendix S1) [6]. The senior radiologist re-evaluated 50 randomly selected participants after 2 months to assess intra-observer repeatability.

Additionally, the background parenchymal enhancement (BPE) levels were categorized as minimal, mild, moderate, or marked in the breast containing the lesion according to the BI-RADS lexicon [4].

### Clinicopathologic data collection

We collected clinicopathologic data, including age, menopausal status, histopathological type, estrogen/progesterone receptor (ER/PR) status, human epidermal growth factor receptor 2 (HER2) status, Ki-67 status, molecular subtype, pCR status (specific criteria are detailed in Appendix S1), and lymph node metastasis.

### Statistical analysis

This study aims to evaluate the noninferiority of semi-quantitative parameters compared to quantitative parameters. The difference in areas under the receiver operating characteristic (ROC) curves (AUC) (∆AUC) served as the primary metric for this comparison, with statistical significance evaluated using the DeLong test. Noninferiority was concluded if the lower bound of the 95% confidence interval (CI) for ∆AUC between semi-quantitative and quantitative parameters exceeds −0.05. Should noninferiority be demonstrated, the superiority of semi-quantitative parameters was further tested, with superiority claimed if the lower bound of the 95% CI for ∆AUC exceeds 0. For the power analysis, assuming a target AUC of 0.85, 80% power, 5% type I error, 20% type II error, a noninferiority margin of 0.05, and a 1:4 ratio of negative to positive cases, the estimated sample size was approximately 110.

For statistical analysis, BPE level was dichotomized into low-BPE (minimal/mild) and high-BPE (moderate/marked) subgroups [15, 16]. Continuous variables were assessed for normality using the Kolmogorov-Smirnov test. Normally distributed data were compared using the Student’s *t*-test, while non-normally distributed data were analyzed with the Wilcoxon–Mann–Whitney test. The Pearson correlation coefficient (r) was used to assess correlations between continuous variables. Inter- and intra-observer reliability for these parameters was examined using the intraclass correlation coefficient (ICC). The statistical analyses were conducted by one co-author (Y.H.) using R (version 4.3.1) and MedCalc (version 23.0.6). Statistical significance was defined as *p* < 0.05, with all tests being two-sided.

## Results

### Participant characteristics

A total of 359 lesions (69 benign lesions and 290 malignant lesions) were identified in 339 participants, with 20 participants presenting bilateral lesions. The baseline characteristics are summarized in Table 1. Malignant lesions were associated with older age, postmenopausal status, and larger tumor diameter than benign lesions (all *p* < 0.001). The malignant lesions comprised 71 (24%) luminal A, 121 (42%) luminal B, 48 (17%) TNBC, and 50 (17%) HER2-enriched subtypes. Among malignancies, 144 participants received complete NAC, with pCR assessed postoperatively. The overall pCR rate was 24% (34/144), and this variable was associated with ER negativity, PR negativity, HER2 positivity, molecular subtype, and lymph node negativity (all *p* < 0.05).

**Table 1:** Clinicopathologic characteristics of participants with breast lesions

Characteristics	Group					
	Benignancy (*n* = 69)	Malignancy (*n* = 290)	*p*-value	pCR (n* = 34)	non-pCR (n* = 110)	*p*-value
Age (years)	43 ± 11 (22–68)	52 ± 9 (22–78)	**< 0.001**	50 ± 8 (22–69)	50 ± 9 (22–70)	0.94
Menopausal status			**< 0.001**			0.84
Premenopausal	57 (83)	127 (44)		16 (47)	56 (51)	
Postmenopausal	12 (17)	163 (56)		18 (53)	54 (49)	
Benign lesions type			/			/
Fibroadenoma	34 (49)	/		/	/	
Adenosis	22 (32)	/		/	/	
Mastitis	6 (9)	/		/	/	
Papilloma	4 (6)	/		/	/	
Phyllodes tumor	3 (4)	/		/	/	
Malignant lesions type			/			0.71
Invasive ductal carcinoma	/	260 (90)		33 (97)	107 (97)	
Ductal carcinoma in situ	/	14 (5)		1 (3)	1 (1)	
Mucinous carcinoma	/	7 (2)		0 (0)	1 (1)	
Invasive lobular carcinoma	/	6 (2)		0 (0)	0 (0)	
Invasive micropapillary carcinoma	/	3 (1)		0 (0)	1 (1)	
Estrogen receptor status			/			**< 0.001**
Positive	/	187 (64)		10 (29)	77 (70)	
Negative	/	103 (36)		24 (71)	33 (30)	
Progesterone receptor status			/			**< 0.001**
Positive	/	128 (44)		3 (9)	52 (47)	
Negative	/	162 (56)		31 (91)	58 (53)	
HER2 status			/			**0.001**
Positive	/	104 (36)		22 (65)	34 (31)	
Negative	/	186 (64)		12 (35)	76 (69)	
Ki-67 status			/			0.39
Positive	/	181 (62)		25 (73)	70 (64)	
Negative	/	109 (38)		9 (27)	40 (36)	
Molecular subtype			/			**0.01**
Luminal A	/	71 (24)		1 (3)	30 (27)	
Luminal B	/	121 (42)		11 (32)	49 (45)	
TNBC	/	48 (17)		8 (24)	17 (15)	
HER2-enriched		50 (17)		14 (41)	14 (13)	
Lymph node metastasis			/			**0.02**
Positive	/	185 (64)		23 (68)	96 (87)	
Negative	/	105 (36)		11 (32)	14 (13)	
Maximum diameter (cm)	1.8 ± 1.3	3.6 ± 1.9	**< 0.001**	3.5 ± 1.6	3.9 ± 1.8	0.35

Unless otherwise specified, categorical data are numbers of lesions, with percentages in parentheses. Age is expressed as mean ± standard deviation, with the range in parentheses

*p*-values were analyzed using the Wilcoxon–Mann–Whitney test, Chi-square test, and Fisher Exact Test

n* represents the number of participants

*ER* estrogen receptor, *PR* progesterone receptor, *HER2* human epidermal growth factor receptor 2, *TNBC* triple-negative breast cancer, *pCR* pathologic complete response

Bold values indicate statistical significance *p* < 0.05

### Semi-quantitative and quantitative parameter differences between benign and malignant lesions

As shown in Table 2, all five semi-quantitative parameters demonstrated significant differences between benign and malignant breast lesions. Specifically, the mean values of WIS (0.5 ± 0.2 vs 1.1 ± 0.4%/s, *p* < 0.001), PEI (0.4 ± 0.2 vs 0.7 ± 0.3, *p* < 0.001), and iAUC (0.2 ± 0.1 vs 0.4 ± 0.1, *p* < 0.001) were lower in benign lesions than in malignant lesions. In contrast, TTP (48.0 ± 17.1 vs 39.8 ± 11.3 s, *p* < 0.001) and BAT (20.2 ± 11.2 vs 13.6 ± 7.4 s, *p* < 0.001) were higher in benign lesions than in malignant lesions.

**Table 2:** Comparison of semi-quantitative and quantitative parameters for different pathologic types, molecular subtypes, and pathologic complete response to neoadjuvant chemotherapy

Group	Semi-quantitative					Quantitative		
	WIS (%/s)	TTP (s)	BAT (s)	PEI	iAUC	*K*^*trans*^ (min^−^^1^)	*k*_*ep*_ (min^−^^1^)	*v*_*e*_
Pathologic type								
Benignancy (*n* = 69)	0.5 ± 0.2	48.0 ± 17.1	20.2 ± 11.2	0.4 ± 0.2	0.2 ± 0.1	0.3 ± 0.1	0.4 ± 0.2	0.7 ± 0.5
Malignancy (*n* = 290)	1.1 ± 0.4	39.8 ± 11.3	13.6 ± 7.4	0.7 ± 0.3	0.4 ± 0.1	0.5 ± 0.3	1.0 ± 0.5	0.6 ± 0.3
*p*-value	**< 0.001**	**< 0.001**	**< 0.001**	**< 0.001**	**< 0.001**	**< 0.001**	**< 0.001**	**< 0.001**
Molecular subtype								
Luminal A (*n* = 71)	1.0 ± 0.4	40.1 ± 12.7	13.3 ± 7.0	0.8 ± 0.3	0.4 ± 0.2	0.6 ± 0.3	0.9 ± 0.5	0.6 ± 0.3
Luminal B (*n* = 121)	1.1 ± 0.4	40.0 ± 11.1	12.8 ± 7.5	0.7 ± 0.3	0.4 ± 0.1	0.5 ± 0.3	1.0 ± 0.5	0.5 ± 0.2
TNBC (*n* = 48)	1.1 ± 0.4	39.5 ± 12.1	14.2 ± 8.2	0.7 ± 0.3	0.4 ± 0.1	0.5 ± 0.3	1.0 ± 0.5	0.5 ± 0.2
HER2-enriched (*n* = 50)	1.1 ± 0.4	36.6 ± 8.2	15.3 ± 7.2	0.7 ± 0.4	0.4 ± 0.2	0.5 ± 0.2	0.9 ± 0.3	0.6 ± 0.3
*p*-value	0.75	0.23	0.21	0.12	0.17	0.65	0.24	0.07
Pathological assessment of response								
pCR (*n* = 50)	1.1 ± 0.4	38.3 ± 8.0	15.3 ± 7.6	0.8 ± 0.4	0.4 ± 0.2	0.5 ± 0.2	0.9 ± 0.3	0.6 ± 0.3
Non-pCR (*n* = 94)	1.1 ± 0.4	39.1 ± 11.6	13.7 ± 7.7	0.7 ± 0.3	0.4 ± 0.1	0.5 ± 0.3	1.0 ± 0.5	0.6 ± 0.2
*p*-value	0.54	0.83	0.26	0.39	0.43	0.90	0.16	0.18

Data are means ± standard deviations. *p* values were analyzed using the Wilcoxon-Mann-Whitney, Kruskal-Wallis test, and Student’s t-tests according to distribution normality. *WIS* wash-in slope, *TTP* time-to-peak, *BAT* bolus arrival time, *iAUC* initial area under the curve in 60 sec, *K*^*trans*^ volume transfer constant, *k*_*ep*_ rate constant, *v*_*e*_ extravascular extracellular space volume, *HER2* human epidermal growth factor 2, *TNBC* triple-negative breast cancer, *pCR* pathologic complete response

Bold values indicate statistical significance *p* < 0.05

**Fig. 3:**
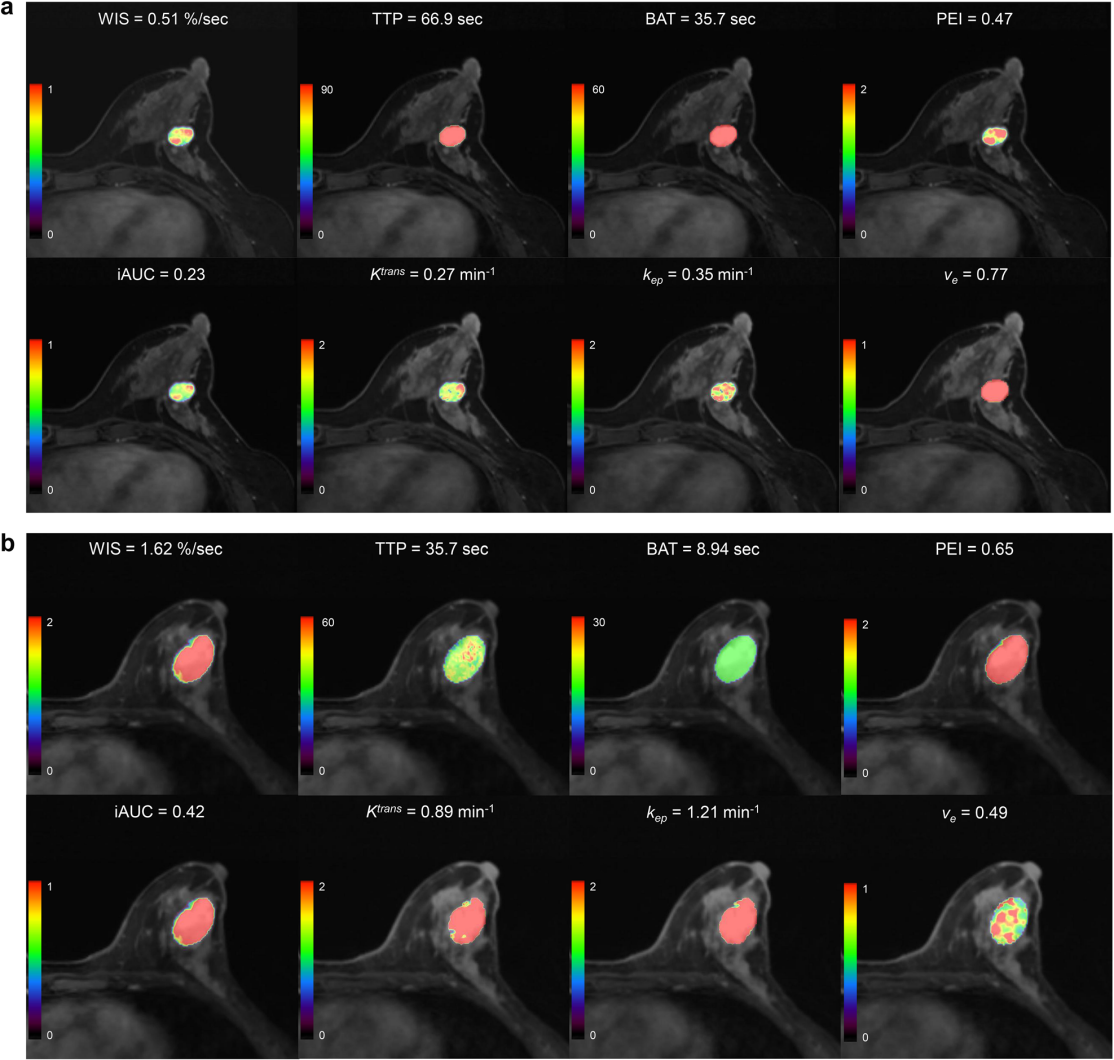
Colormaps of semi-quantitative and quantitative parameters. **a** A 35-year-old female participant with a pathologically confirmed benign fibroadenoma in the left breast. **b** A 40-year-old woman with pathologically confirmed invasive ductal carcinoma in the left breast. WIS, wash-in slope; TTP, time-to-peak; BAT, bolus arrival time; PEI, peak enhancement intensity; iAUC, initial area under the curve in 60 s; *K*^*trans*^, volume transfer constant; *k*_*ep*_, rate constant; *v*_*e*_, extravascular extracellular space volume

Furthermore, quantitative parameters also showed significant differences between benign and malignant breast lesions (Supplementary Fig. S1). The mean *K*^*trans*^ (0.3 ± 0.1 vs 0.5 ± 0.3 min^−^^1^, *p* < 0.001) and *k*_*ep*_ (0.4 ± 0.2 vs 1.0 ± 0.5 min^−^^1^, *p* < 0.001) were lower in benign lesions than in malignant lesions, while *v*_*e*_ was higher in benign lesions than in malignant lesions (0.7 ± 0.5 vs 0.6 ± 0.3, *p* < 0.001). Examples of malignant and benign findings are presented in Fig. 3a, b, along with their colormaps for semi-quantitative and quantitative parameters. Subgroup analysis by BPE level confirmed significant differences for most parameters between benign and malignant lesions in both low- and high-BPE subgroups (all *p* < 0.05; Supplementary Table S3), with the exception of *v*_*e*_ in the low-BPE and of TTP and BAT in the high-BPE subgroup (all *p* > 0.05).

### Semi-quantitative and quantitative parameter differences among molecular subtypes and pCR vs non-pCR groups, and pathologic prognostic factors

Neither semi-quantitative nor quantitative parameters revealed differences among four molecular subtypes (luminal A, luminal B, TNBC, and HER2-enriched) (Supplementary Fig. S2A) or between pCR and non-pCR groups (Supplementary Fig. S2B) (all *p* > 0.05). This lack of significant differences in these parameters among four molecular subtypes or between pCR and non-pCR groups was generally consistent when stratified by BPE level (all *p* > 0.05; Supplementary Table S3), though significant differences in iAUC and *v*_*e*_ (*p* = 0.03 and 0.04, respectively) between pCR and non-pCR groups emerged within the high-BPE subgroup. Similarly, no significant differences were observed in any semi-quantitative and quantitative parameters across pathologic prognostic factors (ER, PR, HR, and HER2 status, Ki-67 index, nuclear grade, tumor size, and lymph node status) or clinical factors (menopausal status) (all *p* > 0.05), except for significant differences in TTP between ER-positive and ER-negative (*p* = 0.04) and between HR-positive and HR-negative groups (*p* = 0.05). Additionally, significant differences were observed in *k*_*ep*_ (*p* = 0.03) and *v*_*e*_ (*p* = 0.045) between PR-positive and PR-negative groups (Supplementary Table S4).

### Correlations between semi-quantitative and quantitative parameters

**Fig. 4:**
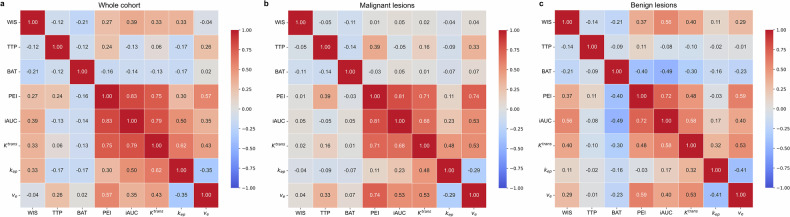
Correlation heatmap of semi-quantitative and quantitative parameters. **a** Correlation matrix for all DCE MRI parameters in the total cohort. **b** Correlation matrix for benign breast lesions. **c** Correlation matrix for malignant breast lesions. WIS, wash-in slope; TTP, time-to-peak; BAT, bolus arrival time; PEI, peak enhancement intensity; iAUC, initial area under the curve in 60 s; *K*^*trans*^, volume transfer constant; *k*_*ep*_, rate constant; *v*_*e*_, extravascular extracellular space volume

Figure 4 presents correlation heatmaps between semi-quantitative and quantitative parameters. For semi-quantitative parameters, WIS showed positive correlations with PEI and iAUC, while TTP and BAT exhibited negative correlations with other parameters (all *p* < 0.05). Among quantitative parameters, *K*^*trans*^ positively correlated with *k*_*ep*_ (r = 0.62, *p* < 0.001). Notably, WIS exhibited positive correlations with both *K*^*trans*^ and *k*_*ep*_ (r = 0.33, *p* < 0.001 for both), but not with *v*_*e*_ (r = −0.04, *p* = 0.49). TTP inversely correlated with *k*_*ep*_ (r = −0.17, *p* < 0.001) while positively correlated with *v*_*e*_ (r = 0.26, *p* < 0.001). BAT showed negative correlations with both *K*^*trans*^ and *k*_*ep*_ (r = −0.13 and −0.17; both *p* < 0.001). A strong positive correlation was observed between PEI and *K*^*trans*^ (r = 0.75, *p* < 0.001), and between iAUC and *K*^*trans*^ (r = 0.79, *p* < 0.001). PEI also demonstrated a moderate correlation with *v*_*e*_ (r = 0.57, *p* < 0.001), as did iAUC and *k*_*ep*_ (r = 0.50, *p* < 0.001) (Supplementary Table S5).

### Comparison of diagnostic performance between semi-quantitative and quantitative parameters in differentiating benign and malignant breast lesions

**Fig. 5:**
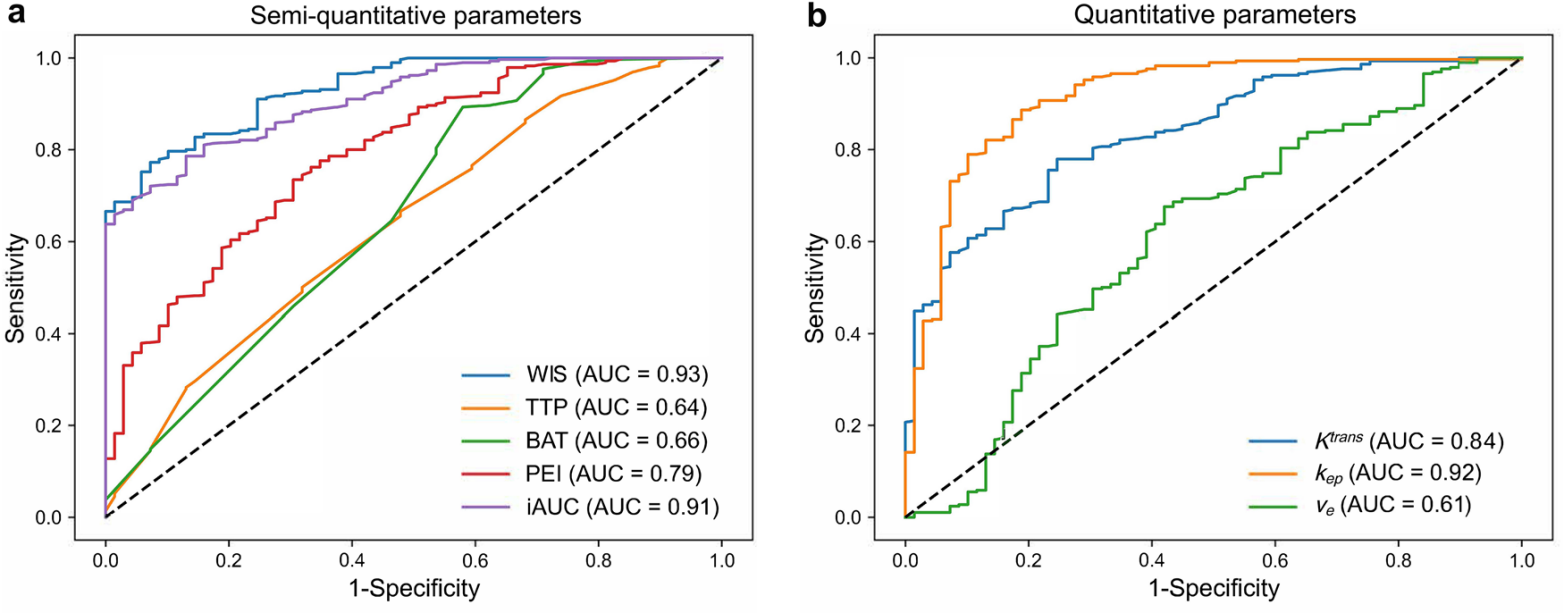
Receiver operating characteristic (ROC) curves for (**a**) semi-quantitative parameters and (**b**) quantitative parameters. WIS, wash-in slope; TTP, time-to-peak; BAT, bolus arrival time; PEI, peak enhancement intensity; iAUC, initial area under the curve in 60 s; *K*^*trans*^, volume transfer constant; *k*_*ep*_, rate constant; *v*_*e*_, extravascular extracellular space volume

The diagnostic performance metrics for five semi-quantitative parameters and three quantitative parameters are summarized in Table 3, with the corresponding ROC curves presented in Fig. 5. Regarding the semi-quantitative parameters, the AUC for WIS was 0.93 (95% CI: 0.90, 0.96), while the AUCs for TTP, BAT, PEI, and iAUC were 0.64 (95% CI: 0.57, 0.72), 0.66 (95% CI: 0.58, 0.74), 0.79 (95% CI: 0.73, 0.85), and 0.91 (95% CI: 0.87, 0.94), respectively, indicating that the AUC for WIS was higher than AUCs for TTP, BAT, PEI, and iAUC (∆AUC: 0.27, 0.28, 0.15, and 0.03; all *p* < 0.001). Additionally, the AUC for iAUC was greater than TTP, BAT, and PEI (∆AUC: 0.27, 0.25, and 0.12; all *p* < 0.001). No significant difference was observed between the AUCs for TTP and BAT (∆AUC: 0.02, *p* = 0.73) (Table 4).

**Table 3:** Performance measures of semi-quantitative and quantitative parameters in differentiating benign and malignant lesions

Parameter	AUC	Cut-off value	Sensitivity (%)	Specificity (%)	PPV (%)	NPV (%)	Accuracy (%)
Semi-quantitative							
WIS (%/s)	0.93 [0.90, 0.96]	0.79	77 [72, 82] (224/290)	93 [86, 98] (64/69)	98 [96, 100] (224/229)	49 [40, 58] (64/130)	80 [76, 84] (288/359)
TTP (s)	0.64 [0.57, 0.72]	42.36	67 [61, 72] (193/290)	52 [41, 65] (36/69)	85 [81, 90] (193/226)	27 [20, 35] (36/133)	64 [59, 69] (229/359)
BAT (s)	0.66 [0.58, 0.74]	22.32	89 [86, 93] (259/290)	42 [30, 53] (29/69)	87 [83, 90] (259/299)	48 [35, 62] (29/60)	80 [76, 84] (288/359)
PEI	0.79 [0.73, 0.85]	0.51	74 [68, 78] (213/290)	70 [59, 81] (48/69)	91 [87, 94] (213/234)	38 [30, 48] (48/125)	73 [68, 77] (261/359)
iAUC	0.91 [0.87, 0.94]	0.28	79 [73, 83] (228/290)	87 [79, 95] (60/69)	96 [94, 98] (228/237)	49 [40, 58] (60/122)	80 [75, 84] (288/359)
Quantitative							
* K*^*trans*^ (min^−^^1^)	0.84 [0.79, 0.89]	0.31	78 [73, 83] (226/290)	75 [65, 85] (52/69)	93 [89, 96] (226/243)	45 [36, 54] (52/116)	77 [73, 82] (278/359)
* k*_*ep*_ (min^−^^1^)	0.92 [0.87, 0.95]	0.56	89 [85, 92] (257/290)	81 [71, 90] (56/69)	95 [92, 97] (257/270)	63 [53, 73] (56/89)	87 [83, 90] (313/359)
*v*_*e*_	0.61 [0.53, 0.69]	0.59	68 [62, 73] (196/290)	58 [47, 69] (40/69)	87 [82, 91] (196/225)	30 [22, 38] (40/134)	66 [61, 70] (236/359)

Unless otherwise specified, data are percentages, followed by 95% CI in brackets and proportions in parentheses. AUCs are expressed as decimal numbers followed by 95% CI in parentheses

*AUC* area under the receiver operating characteristic curve, *PPV* positive predictive value, *NPV* negative predictive value, *WIS* wash-in slope, *TTP* time-to-peak, *BAT* bolus arrival time, *iAUC* initial area under the curve in 60 s, *K*^*trans*^ volume transfer constant, *k*_*ep*_ rate constant, *v*_*e*_ extravascular extracellular space volume, *CI* confidence interval

**Table 4:** Differences in the area under the receiver operating characteristic curve between semi-quantitative and quantitative parameters in differentiating benign and malignant breast lesions

Parameter	WIS (%/s)	TTP (s)	BAT (s)	PEI	iAUC	*K*^*trans*^ (min^−^^1^)	*k*_*ep*_ (min^−^^1^)	*v*_*e*_
Semi-quantitative								
WIS (%/s)	/	0.30 (0.22, 0.37)**	0.28 (0.20, 0.35)**	0.18 (0.10, 0.20)**	0.11 (0.07, 0.15)**	0.10 (0.06, 0.14)**	0.02 (−0.02, 0.07)	0.32 (0.23, 0.41)**
TTP (s)	0.30 (0.22, 0.37)**	/	0.02 (−0.10, 0.14)	0.15 (0.04, 0.26)*	0.19 (0.11, 0.27)**	0.20 (0.11, 0.29)**	0.27 (0.19, 0.36)**	0.03 (−0.08, 0.13)
BAT (s)	0.28 (0.20, 0.35)**	0.02 (−0.10, 0.14)	/	0.13 (0.04, 0.21)*	0.17 (0.09, 0.25)**	0.18 (0.09, 0.26)**	0.25 (0.17, 0.34)**	0.05 (−0.08, 0.17)
PEI	0.18 (0.10, 0.20)**	0.15 (0.04, 0.26)*	0.13 (0.04, 0.21)*	/	0.04 (−0.02, 0.11)	0.05 (−0.01, 0.11)	0.13 (0.052, 0.20)**	0.18 (0.06, 0.30)*
iAUC	0.11 (0.07, 0.15)**	0.19 (0.11, 0.27)**	0.17 (0.09, 0.25)**	0.04 (−0.02, 0.11)	/	0.07 (0.03, 0.11)**	0.01 (−0.045, 0.06)	0.30 (0.20, 0.39)**
Quantitative								
*K*^*trans*^ (min^−^^1^)	0.10 (0.06, 0.14)**	0.20 (0.11, 0.29)**	0.18 (0.09, 0.26)**	0.05 (−0.01, 0.11)	0.07 (0.03, 0.11)**	/	0.08 (0.02, 0.13)*	0.23 (0.11, 0.34)**
*k*_*ep*_ (min^−^^1^)	0.02 (−0.02, 0.07)	0.27 (0.19, 0.36)**	0.25 (0.17, 0.34)**	0.13 (0.052, 0.20)**	0.01 (−0.045, 0.06)	0.08 (0.02, 0.13)*	/	0.30 (0.23, 0.37)**
*v*_*e*_	0.32 (0.23, 0.41)**	0.03 (−0.08, 0.13)	0.05 (−0.08, 0.17)	0.18 (0.06, 0.30)*	0.30 (0.20, 0.39)**	0.23 (0.11, 0.34)**	0.30 (0.23, 0.37)**	/

Data are expressed as decimal numbers, with a 95% CI in parentheses. *p*-values were compared using the DeLong test. “**” denotes a *p*-value of < 0.001, while “*” denotes a *p*-value < 0.05

*AUC* area under the receiver operator characteristic curve, *WIS* wash-in slope, *TTP* time-to-peak, *BAT* bolus arrival time, *iAUC* initial area under the curve in 60 s, *K*^*trans*^ volume transfer constant, *k*_*ep*_ rate constant, *v*_*e*_ extravascular extracellular space volume, *CI* confidence interval

For the quantitative parameters, the AUCs for *k*_*ep*_, *K*^*trans*^, and *v*_*e*_ were 0.92 (95% CI: 0.87, 0.95), 0.84 (95% CI: 0.79, 0.89), and 0.61 (95% CI: 0.53, 0.69), respectively. The AUCs for both *k*_*ep*_ and *K*^*trans*^ were higher than those for *v*_*e*_ (∆AUC: 0.30 and 0.23; both *p* < 0.001). Furthermore, the AUC for *k*_*ep*_ was greater than that for *K*^*trans*^ (∆AUC: 0.08; *p* < 0.05) (Table 4).

Among all the parameters, WIS achieved the highest AUC, which was higher than the AUCs for both *K*^*trans*^ and *v*_*e*_ (∆AUC: 0.10 [95% CI: 0.06, 0.14] and 0.32 [95% CI: 0.23, 0.41]; both *p* < 0.001). However, no evidence of a difference in the AUC was found between WIS and *k*_*ep*_ (∆AUC: 0.02 [95% CI: −0.02, 0.07]; *p* = 0.35) or between iAUC and *k*_*ep*_ (∆AUC: 0.01 [95% CI: −0.045, 0.06]; *p* = 0.83) (Table 4). The comparison between semi-quantitative parameters and quantitative parameters met the predefined noninferiority criterion. Subgroup analysis stratified by BPE level demonstrated generally consistent diagnostic performance across parameters (Supplementary Tables S8–S10), with WIS and *k*_*ep*_ maintaining their superior diagnostic performance in both low- and high-BPE subgroups.

### Repeatability and reproducibility of semi-quantitative and quantitative parameters

**Fig. 6:**
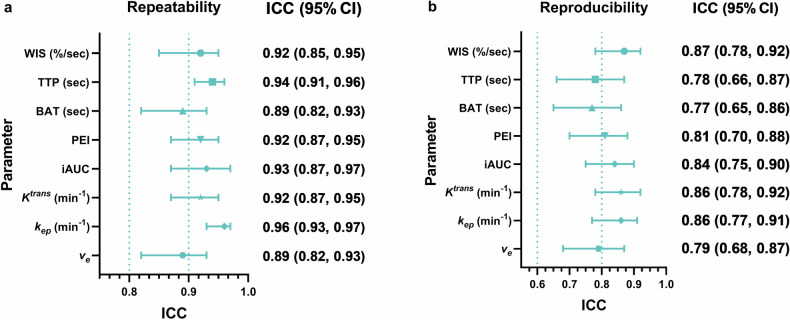
Forest plot illustrating (**a**) intra-observer repeatability and (**b**) inter-observer reproducibility of semi-quantitative parameters versus quantitative parameters. WIS, wash-in slope; TTP, time-to-peak; BAT, bolus arrival time; PEI, peak enhancement intensity; iAUC, initial area under the curve in 60 s; *K*^*trans*^, volume transfer constant; *k*_*ep*_, rate constant; *v*_*e*_, extravascular extracellular space volume

The repeatability of the assessment was excellent for WIS, TTP, PEI, iAUC, *K*^*trans*^, and *k*_*ep*_ (ICCs = 0.92, 0.94, 0.92, 0.93, 0.92, and 0.96, respectively) and good for BAT and *v*_*e*_ (both ICCs = 0.89). The reproducibility between the two readers was good for both semi-quantitative and quantitative parameters, with ICCs of 0.87 (WIS), 0.78 (TTP), 0.77 (BAT), 0.81 (PEI), 0.84 (iAUC), 0.86 (*K*^*trans*^), 0.87 (*k*_*ep*_), and 0.79 (*v*_*e*_) (Fig. 6 and Supplementary Table S11).

## Discussion

UF-DCE MRI, characterized by its short scan duration (≤ 2 min) and model-free analysis, is increasingly recognized for its potential in breast cancer screening and diagnosis [15, 17]. This study employed a high-temporal-resolution DCE MRI acquisition to directly compare the performance of semi-quantitative parameters from the early-phase UF-DCE dataset with quantitative parameters derived from the full time-course UF-DCE dataset in the differential diagnosis, molecular subtyping, and treatment prediction of breast cancer in the same cohort. Our findings demonstrated that semi-quantitative parameters exhibited noninferior diagnostic performance compared with quantitative parameters for differentiating benign and malignant breast lesions. The AUCs for two semi-quantitative parameters, WIS and iAUC, were not different from the quantitative parameter, *k*_*ep*_ (WIS: AUC = 0.93 vs 0.92, ∆AUC: 0.02 [95% CI: −0.02, 0.07], *p* = 0.35; iAUC: AUC = 0.91 vs 0.92, ∆AUC: 0.01 [95% CI: −0.045, 0.05]; *p* = 0.83). However, neither semi-quantitative nor quantitative parameters effectively predicted molecular subtypes or pCR.

We observed that WIS, PEI, and iAUC values were significantly higher in malignancies than in benign lesions, while the TTP and BAT values were lower (all *p* < 0.001). These results aligned with previous studies and reflect the key hemodynamic characteristics of malignancies, including increased angiogenesis, vascular permeability, and perfusion [18–21]. Derived from the time-signal intensity curve, these semi-quantitative parameters are highly reproducible and effectively capture these pathophysiological features associated with early, rapid contrast agent uptake, making them reliable in differentiating benign from malignant lesions.

Similarly, quantitative parameters, including *K*^*trans*^, *k*_*ep*_, and *v*_*e*_, also exhibit significant differences between benignancies and malignancies, consistent with their roles in characterizing vascular permeability and extracellular space [22]. Benign lesions tend to have fewer neovessels with intact endothelial linings, leading to slower contrast agent extravasation and prolonged washout, resulting in lower *K*^*trans*^ and *k*_*ep*_ values [23]. Our findings are consistent with previous studies reporting that both *K*^*trans*^ and *k*_*ep*_ values of malignancies are significantly higher than those of benign lesions [5, 24]. Interestingly, we noted that *v*_*e*_ values were significantly lower in malignant lesions, likely due to the higher cellular and microvascular density, which reduces interstitial space [25]. However, discrepancies exist in the literature regarding *v*_*e*_ values, with some studies reporting higher values in malignancies, while others observe the opposite [5, 26]. Further validation through large-scale studies is essential to confirm these findings.

We further explored the correlations between semi-quantitative and quantitative parameters, revealing strong positive correlations between PEI and *K*^*trans*^ (r = 0.75, *p* < 0.001) and iAUC and *K*^*trans*^ (r = 0.79, *p* < 0.001), as well as moderate correlations between PEI and *v*_*e*_ (r = 0.57, *p* < 0.001) and iAUC and *k*_*ep*_ (r = 0.50, *p* < 0.001). These findings suggest that semi-quantitative parameters reflect similar physiological processes as quantitative parameters, particularly regarding tumor vascularity and permeability. The strong correlations of PEI and iAUC with *K*^*trans*^ reflect enhanced contrast agent diffusion efficiency in tumors with hyperperfusion and elevated vascular permeability, consistent with the pathological angiogenesis patterns in malignancies [27]. The moderate PEI-*v*_*e*_ and iAUC-*k*_*ep*_ correlation suggests that high blood flow and permeability in malignant lesions may coexist with smaller interstitial spaces, where rapid contrast accumulation is followed by accelerated washout due to leaky neovasculature [11, 28].

Despite its advantages in the differential diagnosis of breast cancer, our study revealed that neither early-phase nor full time-course UF-DCE MRI effectively distinguished molecular subtypes or predicted pCR. The absence of differences in UF-DCE parameters across molecular subtypes and between pCR and non-pCR groups may stem from overlapping vascular characteristics, particularly in aggressive subtypes such as HER2-enriched and TNBC. These subtypes exhibit distinct receptor expression and proliferative activity but may have similar perfusion patterns due to pronounced angiogenesis [29]. Furthermore, pre-treatment DCE MRI may insufficiently resolve spatiotemporal heterogeneity and therapeutic response patterns, while longitudinal monitoring of early perfusion changes during NAC may hold greater predictive value [30]. However, Ramtohul et al have suggested WIS based on pre-treatment UF-DCE MRI as a predictive marker for pCR following NAC in locally advanced breast cancer in a very limited cohort [31]. Discrepancies with previous studies could be attributed to variations in patient cohorts, imaging protocols, or analytical approaches [31, 32]. Future research should focus on exploring multimodal imaging integration, longitudinal treatment monitoring, and AI-driven radiomics approaches to enhance the accuracy of molecular subtyping and treatment response prediction.

Our findings confirmed that the diagnostic performance of WIS is noninferior to that of *k*_*ep*_ in differentiating malignancies from benign ones, supporting the potential of early-phase UF-DCE as a time-efficient alternative to full time-course UF-DCE modeling for breast lesion classification. Given its shorter scan duration and the simplicity and objectivity of its model-free analysis compared to full time-course UF-DCE modeling, early-phase UF-DCE MRI may offer greater clinical accessibility and broader applicability. Our BPE subgroup analysis further reinforces the clinical applicability of early-phase UF-DCE MRI.

This study had several limitations. First, it is a single-center, small-sample study, and the findings require validation in larger, multicenter cohorts. Second, Task 3 analysis was based solely on pre-treatment DCE MRI data obtained at a single time point, which may have contributed to the negative results. Future studies should incorporate longitudinal DCE MRI data from multiple time points to explore the feasibility of DCE MRI in predicting pCR. Third, the sample distribution was imbalanced, with a predominance of malignant lesions due to the study population being limited to cases with clinical indications for breast MRI. Future large-scale studies with more balanced lesion distributions are needed to confirm these findings.

In conclusion, early-phase UF-DCE MRI demonstrated noninferiority to full time-course UF-DCE MRI in differentiating benign and malignant breast lesions, suggesting its potential as a viable alternative for breast lesion classification in further clinical applications. However, its potential for molecular subtyping and treatment response prediction of breast cancer requires further large-scale multicenter validation.

## Supplementary information

ELECTRONIC SUPPLEMENTARY MATERIAL

## Data Availability

The dataset used or analyzed during the current study is available from the corresponding author on reasonable request.

## Abbreviations

AUC: Area under the receiver operating characteristic curve
BAT: Bolus arrival time
DCE: Dynamic contrast-enhanced
iAUC: Initial area under the curve in 60 s
K^trans^: Volume transfer constant, *k*_*ep*_ rate constant
PEI: Peak enhancement intensity
TTP: Time-to-peak, *v*_*e*_ extravascular extracellular space volume
WIS: Wash-in slope
