# Publisher Correction: Early-phase semi-quantitative analysis versus full time-course quantitative modeling of ultrafast dynamic contrast-enhanced MRI for breast cancer diagnosis, molecular subtyping, and treatment response prediction

**DOI:** 10.1186/s13244-026-02233-4

**Published:** 2026-03-02

**Authors:** Ying Cao, Xueqin Gong, Yao Huang, Huifang Chen, Jie Fang, Lu Wang, Lan Li, Sun Tang, Ting Yin, Xiaoxia Wang, Jiuquan Zhang

**Affiliations:** 1 School of Medicine, Chongqing University, Chongqing, China; 2 Department of Radiology, Chongqing University Cancer Hospital, Chongqing Key Laboratory for Intelligent Oncology in Breast Cancer (iCQBC), Chongqing, China; 3 MR Collaborations, Siemens Healthineers Ltd., Chengdu, China

**Correction to: Insights into Imaging**

10.1186/s13244-025-02166-4published online 17 December 2025

The original version of this article has been published with an incorrect Graphical Abstract.

The original publication has been corrected.

