# Supplementary material for: Early-phase semi-quantitative analysis versus full time-course quantitative modeling of ultrafast dynamic contrast-enhanced MRI for breast cancer diagnosis, molecular subtyping, and treatment response prediction
Source: Insights Imaging. 2025 Dec 17;16:279. doi: 10.1186/s13244-025-02166-4 (PMC12712255; doi:10.1186/s13244-025-02166-4)
Supplement: Supplementary file 1 — ELECTRONIC SUPPLEMENTARY MATERIAL [file 13244_2025_2166_MOESM1_ESM.pdf]

Early-phase semi-quantitative analysis versus full time-course quantitative modeling of ultrafast dynamic contrast-enhanced MRI for breast cancer diagnosis, molecular subtyping, and treatment response prediction

ELECTRONIC SUPPLEMENTARY MATERIAL

Appendix S1

1. Histopathological assessment

For participants who underwent upfront surgery, histopathological diagnosis and molecular biomarker status were obtained from the surgical resection specimens. For participants who received neoadjuvant chemotherapy, pretreatment diagnostic core-needle biopsy specimens were used as the baseline pathological reference [1]. Tumors with nuclear staining ≥1% were defined as estrogen receptor (ER)(+)/progesterone receptor (PR)(+), while those with <1% were defined as ER(-)/PR(-) [2]. Human epidermal growth factor receptor 2 (HER2) status was categorized as negative for immunohistochemistry scores of 0 or 1+, and positive for scores of 3+. For 2+ scores, HER2 status was determined by fluorescence in situ hybridization (FISH); amplification indicated HER2(+), while lack of amplification indicated HER2(-) [3; 4]. Additionally, a Ki-67 status threshold of 20% was set, with ≥ 20% denoting high expression and < 20% indicating low expression [5]. Tumors were

categorized into four subtypes as follows: (i) luminal A: ER/PR-positive, HER2-negative, and Ki-67 < 20%; (ii) luminal B: a) ER/PR(+) and HER2(+), b) ER/PR(+), HER2(-), and Ki-67 ≥ 20%; (iii) HER2-enriched: ER/PR(-), HER2(+); and (iv) triple-negative breast cancer (TNBC): ER/PR(-) and HER2(-) [6].

Pathologic complete response (pCR) was defined as the absence of residual invasive cancer in both the breast and axillary lymph nodes (ypT0/is ypN0), with the allowance of residual ductal carcinoma in situ, as determined by surgical histopathological examination.

2. The definition of semi-quantitative and quantitative parameters

A total of 100-phase dynamic contrast-enhanced (DCE) MRI acquisitions were performed with a temporal resolution of 4.5 seconds, covering a total scan duration of 450 seconds. The initial 30 phases (135 sec) were designated as early-phase ultrafast DCE (UF-DCE) MRI, while the entire 100-phase dataset (450 sec) was utilized as full time-course UF-DCE MRI.

Five semi-quantitative parameters were derived from the early-phase UF-DCE MRI dataset, capturing early contrast dynamics without reliance on pharmacokinetic modeling. Meanwhile, three model-based quantitative parameters were computed from the full time-course UF-DCE MRI dataset, providing a quantitative characterization of contrast agent exchange between plasma and tissue compartments.

2.1 Semi-quantitative parameters from early-phase UF-DCE MRI (30 Phases, 135 sec)

Semi-quantitative parameters are generated without the need for complex pharmacokinetic modeling. These parameters are based on the temporal changes in signal intensity during the early phases of contrast agent uptake. The five key semi-quantitative parameters included wash-in slope (WIS) [7], time to peak (TTP) [8], arrival time (BAT) [9], peak enhancement intensity (PEI) [10], and initial area under the curve in 60 sec (iAUC) [11]. Their detailed definitions were as follows:

- **WIS**: Defined as the slope of the contrast enhancement curve between the onset of contrast uptake and the peak intensity within the lesion.
- **TTP**: Represents the time interval between the onset of contrast enhancement and the point of maximum signal intensity within the lesion.
- **BAT**: Defined as the time from the initiation of contrast injection to the onset of measurable enhancement in the lesion.
- **PEI**: Denotes the maximum enhancement intensity reached by the lesion during contrast uptake.
- **iAUC**: Represents the area under the time-intensity curve from BAT to 60 seconds post-injection.

2.2 Quantitative parameters from full time-course UF-DCE MRI (100 Phases, 450 sec)

Quantitative parameters are generated by employing a two-compartment pharmacokinetic model (*Tofts* model), which describe the exchange of contrast agent between the vascular space and the extracellular extravascular space (EES). These parameters provide insights into the microvascular environment and tissue composition. The three key quantitative parameters are as follows [12-14]:

- **Volume transfer constant (K^{trans})**: Represents the rate at which contrast agent transfers from the plasma compartment to the EES, expressed in min^{-1} or mL/min/100mL tissue.
- **Rate constant (k_{ep})**: Describes the efflux rate of contrast agent from the EES back into the plasma, calculated as $k_{ep} = K^{trans}/v_e$, and typically expressed in min^{-1}.
- **Extracellular extravascular space volume fraction (v_e)**: Denotes the fraction of the total tissue volume occupied by the EES.

References

1 Allison KH, Hammond MEH, Dowsett M et al (2020) Estrogen and Progesterone Receptor Testing in Breast Cancer: ASCO/CAP Guideline Update. 38:1346-1366

2 Newman LA, Stark A, Chitale D et al (2017) Association Between Benign Breast Disease in African American and White American Women and Subsequent Triple-Negative Breast Cancer. JAMA oncology 3:1102-1106

3 Tutt A, Tovey H, Cheang MCU et al (2018) Carboplatin in BRCA1/2-mutated and triple-negative breast cancer BRCAness subgroups: the TNT Trial. Nat Med 24:628-637

4 Buisseret L, Pommey S, Allard B et al (2018) Clinical significance of CD73 in triple-negative breast cancer: multiplex analysis of a phase III clinical trial. Ann Oncol 29:1056-1062

5 Gómez-Aleza C, Nguyen B, Yoldi G et al (2020) Inhibition of RANK signaling in breast cancer induces an anti-tumor immune response orchestrated by CD8+ T cells. Nat Commun 11:6335

6 Goldhirsch A, Wood WC, Coates AS, Gelber RD, Thürlimann B, Senn HJ (2011) Strategies for subtypes--dealing with the diversity of breast cancer: highlights of the St. Gallen International Expert Consensus on the Primary Therapy of Early Breast Cancer 2011. Ann Oncol 22:1736-1747

7 Ramtohul T, Tescher C, Vaflard P et al (2022) Prospective Evaluation of Ultrafast Breast MRI for Predicting Pathologic Response after Neoadjuvant Therapies. Radiology 305:565-574

8 Cao Y, Wang X, Shi J et al (2023) Multiple parameters from ultrafast dynamic contrast-enhanced magnetic resonance imaging to discriminate between benign and malignant breast lesions: Comparison with apparent diffusion coefficient. Diagn Interv Imaging 104:275-283

9 Onishi N, Sadinski M, Hughes MC et al (2020) Ultrafast dynamic contrast-enhanced breast MRI may generate prognostic imaging markers of breast cancer. Breast Cancer Res 22:58

10 Cao Y, Wang X, Li L et al (2023) Early prediction of pathologic complete response of breast cancer after neoadjuvant chemotherapy using longitudinal ultrafast dynamic contrast-enhanced MRI. Diagn Interv Imaging 104:605-614

11 Onishi N, Sadinski M, Gibbs P et al (2020) Differentiation between subcentimeter carcinomas and benign lesions using kinetic parameters derived from ultrafast dynamic contrast-enhanced breast MRI. Eur Radiol 30:756-766

12 El Khouli RH, Macura KJ, Kamel IR, Jacobs MA, Bluemke DA (2011) 3-T dynamic contrast-enhanced MRI of the breast: pharmacokinetic parameters versus conventional kinetic curve analysis. AJR Am J Roentgenol 197:1498-1505

13 Wu C, Pineda F, Hormuth DA, 2nd, Karczmar GS, Yankeelov TE (2019) Quantitative analysis of vascular properties derived from ultrafast DCE-MRI to discriminate malignant and benign breast tumors. Magn Reson Med 81:2147-2160

14 Wang W, Lv S, Xun J et al (2022) Comparison of diffusion kurtosis imaging and dynamic contrast enhanced MRI in prediction of prognostic factors and molecular subtypes in patients with breast cancer. Eur J Radiol 154:110392

Table S1: Acquisition parameters for breast MRI

	T1WI	T2WI	DWI	DCE series
Orientation	Axial	Axial	Axial	Axial
Sequence	GRE	TSE	SMS-EPI	CS-VIBE
TR/TE	5.41/2.46	5700/71	4900/51	4.46/1.55
Flip Angle (°)	20	120	/	11
Slice thickness (mm)	1.5	4	2.5	2.5
FOV (mm)	340 × 340	360 × 360	360 × 170	360 × 292
Matrix	352 × 313	448 × 380	170 × 80	384 × 384
Fat-suppression	None	SPAIR	SPAIR	DIXON
Voxel size (mm)	1.0 × 1.0 × 1.5	0.8 × 0.8 × 4.0	2.1 × 2.1 × 2.5	0.9 × 0.9 × 2.5
b-value (sec/mm^2)	/	/	50, 800	/
Acquisition time	59 sec	104 sec	124 sec	450 sec (4.5sec/100 phases)

T1WI T1-weighted imaging, *T2WI* T2-weighted imaging, *DWI* Diffusion-weighted imaging, *DCE* dynamic contrast-enhanced, *GRE* gradient recalled-echo, *TSE* turbo spin-echo, *SMS-EPI* simultaneous multi-slice echo planar imaging, *CS* compressed-sensing, *VIBE* volume-interpolated breath-hold examination, *TR* repetition time, *TE* echo time, *FOV* field of view, *SPAIR* spectral attenuated inversion recovery.

Table S2: Distribution of background parenchymal enhancement in benign and malignant breast lesions

Characteristics	Lesion type		*p* value
	Benign (n = 69)	Malignant (n = 290)	
Background parenchymal enhancement			
Minimal	3 (4.3)	10 (3.4)	
Mild	41 (59.4)	118 (40.7)	< 0.001
Moderate	21 (30.4)	98 (33.8)	
Marked	4 (5.8)	64 (22.1)	

n represents the number of lesions.

Table S3: Comparison of semi-quantitative and quantitative Parameters of UF-DCE MRI for different pathologic types, molecular subtypes, and pathologic complete response to neoadjuvant chemotherapy stratified by background parenchymal enhancement

Group	Semi-quantitative parameter					Quantitative parameter		
	WIS (%/sec)	TTP (sec)	BAT (sec)	PEI	iAUC	K^{trans} (min^{-1})	k_{ep} (min^{-1})	v_e
**Pathologic type**								
**Low-BPE** (n = 172)								
Benignancy (n = 44)	0.4 ± 0.2	49.6 ± 17.7	22.2 ± 12.2	0.4 ± 0.2	0.2 ± 0.1	0.2 ± 0.1	0.4 ± 0.2	0.7 ± 0.5
Malignancy (n = 128)	1.1 ± 0.4	39.5 ± 12.3	14.2 ± 8.1	0.7 ± 0.3	0.4 ± 0.1	0.5 ± 0.3	1.0 ± 0.4	0.5 ± 0.2
p	**< 0.001**	**< 0.001**	**< 0.001**	**< 0.001**	**< 0.001**	**< 0.001**	**< 0.001**	0.12
**High-BPE** (n = 187)								
Benignancy (n = 25)	0.6 ± 0.1	45.1 ± 15.7	16.6 ± 8.5	0.5 ± 0.1	0.2 ± 0.1	0.3 ± 0.1	0.4 ± 0.3	0.8 ± 0.5
Malignancy (n = 162)	1.1 ± 0.4	40.0 ± 10.5	13.1 ± 6.8	0.7 ± 0.3	0.4 ± 0.1	0.5 ± 0.3	1.0 ± 0.5	0.6 ± 0.3
p	**< 0.001**	0.26	0.07	**< 0.001**	**< 0.001**	**< 0.001**	**< 0.001**	**0.003**
**Molecular subtype**								
**Low-BPE** (n = 128)								
Luminal A (n=29)	0.9 ± 0.4	39.5 ± 14.8	14.2 ± 8.2	0.8 ± 0.4	0.4 ± 0.2	0.6 ± 0.4	1.0 ± 0.4	0.6 ± 0.3
Luminal B (n=52)	1.1 ± 0.4	41.4 ± 12.3	13.4 ± 7.5	0.7 ± 0.3	0.4 ± 0.1	0.5 ± 0.3	1.0 ± 0.4	0.5 ± 0.2
TNBC (n=24)	1.1 ± 0.3	40.1 ± 11.4	14.2 ± 8.9	0.6 ± 0.2	0.4 ± 0.1	0.4 ± 0.2	1.0 ± 0.4	0.5 ± 0.2
HER2-enriched (n=23)	1.1 ± 0.4	34.3 ± 8.5	16.2 ± 8.9	0.6 ± 0.3	0.4 ± 0.1	0.4 ± 0.2	0.9 ± 0.3	0.5 ± 0.2
p	0.16	0.20	0.72	0.10	0.16	0.20	0.38	0.25
**High-BPE** (n = 162)								
Luminal A (n=42)	1.1 ± 0.4	40.5 ± 11.2	12.8 ± 6.0	0.8 ± 0.3	0.4 ± 0.1	0.5 ± 0.2	0.9 ± 0.5	0.6 ± 0.2
Luminal B (n=69)	1.0 ± 0.4	40.7 ± 10.2	12.3 ± 7.5	0.7 ± 0.2	0.4 ± 0.1	0.5 ± 0.2	1.1 ± 0.6	0.5 ± 0.2
TNBC (n=24)	1.2 ± 0.5	38.8 ± 12.9	14.2 ± 7.5	0.7 ± 0.4	0.4 ± 0.2	0.6 ± 0.5	1.1 ± 0.6	0.6 ± 0.2
HER2-enriched (n=27)	1.1 ± 0.5	38.5 ± 7.4	14.6 ± 5.3	0.8 ± 0.4	0.5 ± 0.2	0.5 ± 0.2	1.0 ± 0.3	0.6 ± 0.3
p	0.70	0.65	0.23	0.32	0.29	0.88	0.12	0.14
**Pathological assessment of response**								
**Low-BPE** (n = 55)								
pCR (n=13)	1.1 ± 0.5	38.3 ± 8.1	14.7 ± 7.1	0.7 ± 0.2	0.4 ± 0.2	0.4 ± 0.2	0.9 ± 0.2	0.5 ± 0.2
non-pCR (n=42)	1.0 ± 0.4	38.2 ± 11.7	14.3 ± 8.6	0.8 ± 0.4	0.4 ± 0.1	0.6 ± 0.3	1.1 ± 0.5	0.6 ± 0.3
p	0.71	0.93	0.65	0.35	0.12	0.34	0.44	0.75
**High-BPE** (n = 89)								
pCR (n=21)	1.1 ± 0.4	38.3 ± 8.1	15.8 ± 8.0	0.9 ± 0.4	0.5± 0.2	0.5 ± 0.2	0.8 ± 0.3	0.7 ± 0.4
non-pCR (n=68)	1.1 ± 0.4	39.7 ± 11.6	13.3 ± 7.1	0.7 ± 0.2	0.4 ± 0.1	0.5 ± 0.2	1.0 ± 0.5	0.5 ± 0.2
p	0.77	0.95	0.24	0.07	**0.03**	0.14	0.38	**0.04**

Data are means ± SDs. *p* values were analyzed using the Wilcoxon-Mann-Whitney, Kruskal-Wallis test and Student's t-tests according to distribution normality.

UF-DCE ultrafast dynamic contrast-enhanced, *BPE* background parenchymal enhancement, *WIS* wash-in slope, *TTP* time-to-peak, *BAT* bolus arrival time, *iAUC* initial area under the curve in 60 sec, K^{trans} volume transfer constant, k_{ep} rate constant, v_e extravascular extracellular space volume, *HER2* human epidermal growth factor 2, *TNBC* triple-negative breast cancer, *pCR* pathologic complete response.

Table S4: Comparison of semi-quantitative and quantitative parameters for different clinicopathologic prognostic factors and corresponding clinical factors

Group	Semi-quantitative parameters					Quantitative parameters		
	WIS (%/sec)	TTP (sec)	BAT (sec)	PEI	iAUC	K^{trans} (min^{-1})	k_{ep} (min^{-1})	v_e
Premenopausal	1.1 ± 0.4	38.8 ± 10.5	13.1 ± 7.2	0.8 ± 0.3	0.4 ± 0.2	0.5 ± 0.3	0.9 ± 0.4	0.6 ± 0.3
Postmenopausal	1.1 ± 0.4	39.0 ± 11.2	15.1 ± 8.0	0.7 ± 0.3	0.4 ± 0.1	0.5 ± 0.3	1.0 ± 0.5	0.6 ± 0.3
p value	0.72	0.91	0.20	0.90	0.79	0.52	0.58	0.82
Maximum diameter < 2cm	1.0 ± 0.4	36.8 ± 8.5	11.5 ± 7.0	0.8 ± 0.3	0.5 ± 0.2	0.5 ± 0.3	0.9 ± 0.4	0.6 ± 0.2
Maximum diameter ≥ 2cm	1.1 ± 0.4	39.1 ± 11.1	14.3 ± 7.7	0.7 ± 0.3	0.4 ± 0.1	0.5 ± 0.3	1.0 ± 0.4	0.6 ± 0.3
p value	0.25	0.70	0.26	0.69	0.27	0.97	0.77	0.56
Histologic grade (1 or 2)	1.1 ± 0.4	39.1 ± 11.0	13.8 ± 7.3	0.8 ± 0.3	0.4 ± 0.1	0.5 ± 0.3	1.0 ± 0.5	0.6 ± 0.3
Histologic grade (3)	1.2 ± 0.5	39.6 ± 10.2	16.3 ± 10.8	0.6 ± 0.2	0.3 ± 0.1	0.5 ± 0.2	1.1 ± 0.6	0.6 ± 0.4
p value	0.43	0.78	0.62	0.23	0.09	0.84	0.44	0.34
ER positive	1.0 ± 0.4	40.5 ± 11.3	13.8 ± 8.0	0.8 ± 0.3	0.4 ± 0.1	0.5 ± 0.3	1.0 ± 0.5	0.6 ± 0.3
ER negative	1.2 ± 0.5	36.4 ± 9.7	14.5 ± 7.2	0.7 ± 0.3	0.4 ± 0.2	0.5 ± 0.2	1.0 ± 0.4	0.5 ± 0.3
p value	0.06	**0.04**	0.54	0.50	0.64	0.56	0.52	0.18
PR positive	1.1 ± 0.4	38.1 ± 10.5	14.4 ± 8.1	0.7 ± 0.3	0.4 ± 0.1	0.5 ± 0.3	1.0 ± 0.4	0.6 ± 0.3
PR negative	1.1 ± 0.4	40.3 ± 11.3	13.5 ± 6.9	0.7 ± 0.3	0.4 ± 0.1	0.9 ± 0.5	0.9 ± 0.5	0.6 ± 0.2
p value	0.42	0.30	0.58	0.92	0.59	0.55	**0.03**	**0.045**
HR positive	1.0 ± 0.4	40.5 ± 11.3	14.0 ± 8.1	0.8 ± 0.3	0.4 ± 0.1	0.5 ± 0.3	1.0 ± 0.5	0.6 ± 0.3
HR negative	1.2 ± 0.5	36.5 ± 9.8	14.2 ± 7.0	0.7 ± 0.3	0.4 ± 0.2	0.5 ± 0.2	1.0 ± 0.4	0.5 ± 0.3
p value	0.051	**0.05**	0.74	0.51	0.68	0.45	0.62	0.14
HER2 positive	1.1 ± 0.5	37.7 ± 8.5	14.6 ± 7.2	0.8 ± 0.3	0.4 ± 0.2	0.6 ± 0.3	1.0 ± 0.4	0.6 ± 0.3
HER2 negative	1.1 ± 0.4	39.7 ± 12.1	13.8 ± 8.0	0.7 ± 0.3	0.4 ± 0.1	0.5 ± 0.3	0.9 ± 0.4	0.6 ± 0.3
p value	0.39	0.62	0.41	0.41	0.20	0.15	0.19	0.55
Ki-67 positive	1.1 ± 0.4	39.4 ± 10.6	14.1 ± 7.8	0.8 ± 0.3	0.4 ± 0.2	0.5 ± 0.3	1.0 ± 0.4	0.6 ± 0.3
Ki-67 negative	1.1 ± 0.5	36.8 ± 12.0	13.9 ± 7.2	0.7 ± 0.2	0.4 ± 0.1	0.5 ± 0.2	0.9 ± 0.4	0.5 ± 0.2
p value	0.92	0.17	0.92	0.43	0.51	0.46	0.75	0.55
LN metastasis positive	1.1 ± 0.4	38.6 ± 11.5	13.8 ± 7.7	0.7 ± 0.3	0.4 ± 0.1	0.5 ± 0.3	1.0 ± 0.4	0.6 ± 0.2
LN metastasis negative	1.0 ± 0.4	40.4 ± 7.1	15.3 ± 7.4	0.8 ± 0.4	0.5 ± 0.1	0.6 ± 0.3	0.8 ± 0.4	0.7 ± 0.4
p value	0.12	0.12	0.39	0.10	0.18	0.36	0.11	0.21

Data are means ± standard deviations. *p* values were analyzed using the Wilcoxon-Mann-Whitney and Student's t-tests according to distribution normality.
WIS wash-in slope, *TTP* time-to-peak, *BAT* bolus arrival time, *iAUC* initial area under the curve in 60 sec, K^{trans} volume transfer constant, k_{ep} rate constant, v_e extravascular extracellular space volume, *ER* estrogen receptor, *PR* progesterone receptor, *HER2* human epidermal growth factor receptor 2, *HR* hormone receptor, *LN* lymph node.

Table S5: Pearson correlation coefficient for semi-quantitative and quantitative parameters within the whole cohort

Parameter	W_{in} (%/sec)	TTP (sec)	BAT (sec)	PEI	iAUC	K^{trans} (min^{-1})	k_{ep} (min^{-1})	v_e
**Semi-quantitative**								
WIS (%/sec)	/	-0.12*	-0.21**	0.27**	0.39**	0.33**	0.33**	-0.04
TTP (sec)	-0.12*	/	-0.12*	0.24**	-0.13*	0.06	-0.17**	0.26**
BAT (sec)	-0.21**	-0.12*	/	-0.16**	-0.14**	-0.13*	-0.17**	0.02
PEI	0.27**	0.24**	-0.16**	/	0.83**	0.75**	0.30**	0.57**
iAUC	0.39**	-0.13*	-0.14**	0.83**	/	0.79**	0.50**	0.35**
**Quantitative**								
K^{trans} (min^{-1})	0.33**	0.06	-0.13*	0.75**	0.79**	/	0.62**	0.43**
k_{ep} (min^{-1})	0.33**	-0.17**	-0.17**	0.30**	0.50**	0.62**	/	-0.35**
v_e	-0.04	0.26**	0.02	0.57**	0.35**	0.43**	-0.35**	/

Data are expressed as decimal numbers. *p* values were derived from the Student's t-test.

AUC area under the receiver operator characteristic curve, *WIS* wash-in slope, *TTP* time-to-peak, *BAT* bolus arrival time, *iAUC* initial area under the curve in 60 sec, K^{trans} volume transfer constant, k_{ep} rate constant, v_e extravascular extracellular space volume.

**denotes a *p* value of < 0.001, while *denotes a *p* value of < 0.05.

Table S6: Pearson correlation coefficient for UF-DCE semi-quantitative and DCE quantitative parameters in benign lesions

Parameter	WIS (%/sec)	TTP (sec)	BAT (sec)	PEI	iAUC	K^{trans} (min^{-1})	k_{ep} (min^{-1})	v_e
**Semi-quantitative**								
WIS (%/sec)	/	-0.14	-0.21	0.37**	0.56**	0.40**	0.11	0.29*
TTP (sec)	-0.14	/	-0.09	0.11	-0.08	-0.10	-0.02	-0.01
BAT (sec)	-0.21	-0.09	/	-0.40**	-0.49**	-0.30*	-0.16	-0.23
PEI	0.37**	0.11	-0.40**	/	0.72**	0.48**	-0.03	0.59**
iAUC	0.56**	-0.08	-0.49**	0.72**	/	0.58**	0.17	0.40**
**Quantitative**								
K^{trans} (min^{-1})	0.40**	-0.10	-0.30*	0.48**	0.58**	/	0.32**	0.53**
k_{ep} (min^{-1})	0.11	-0.02	-0.16	-0.03	0.17	0.32**	/	-0.41**
v_e	0.29*	-0.01	-0.23	0.59**	0.40**	0.53**	-0.41**	/

Data are expressed as decimal numbers. *p* values were derived from the Student's t-test.

AUC area under the receiver operator characteristic curve, *WIS* wash-in slope, *TTP* time-to-peak, *BAT* bolus arrival time, *iAUC* initial area under the curve in 60 sec, K^{trans} volume transfer constant, k_{ep} rate constant, v_e extravascular extracellular space volume.

**denotes a *p* value of < 0.001, while *denotes a *p* value of < 0.05.

Table S7: Pearson correlation coefficient for UF-DCE semi-quantitative and DCE quantitative parameters in malignant lesions

Parameter	WIS (%/sec)	TTP (sec)	BAT (sec)	PEI	iAUC	K^{trans} (min^{-1})	k_{ep} (min^{-1})	v_e
**Semi-quantitative**								
WIS (%/sec)	/	-0.05	-0.11	0.01	0.05	0.02	-0.04	0.04
TTP (sec)	-0.05	/	-0.14*	0.39**	-0.05	0.16**	-0.09	0.33**
BAT (sec)	-0.11	-0.14*	/	-0.03	0.05	0.01	-0.07	0.07
PEI	0.01	0.39**	-0.03	/	0.81**	0.71**	0.11	0.74**
iAUC	0.05	-0.05	0.05	0.81**	/	0.68**	0.23**	0.53**
**Quantitative**								
K^{trans} (min^{-1})	0.02	0.16**	0.01	0.71**	0.68**	/	0.48**	0.53**
k_{ep} (min^{-1})	-0.04	-0.09	-0.07	0.11	0.23**	0.48**	/	-0.29**
v_e	0.04	0.33**	0.07	0.74**	0.53**	0.53**	-0.29**	/

Data are expressed as decimal numbers. *p* values were derived from the Student's t-test.

AUC area under the receiver operator characteristic curve, *WIS* wash-in slope, *TTP* time-to-peak, *BAT* bolus arrival time, *iAUC* initial area under the curve in 60 sec, K^{trans} volume transfer constant, k_{ep} rate constant, v_e extravascular extracellular space volume.

**denotes a *p* value of < 0.001, while *denotes a *p* value of < 0.05.

Table S8: Performance measures of semi-quantitative and quantitative parameters in differentiating benign and malignant lesions in low- and high-BPE subgroups

Parameter	AUC	Cut-off value	Sensitivity (%)	Specificity (%)	PPV (%)	NPV (%)	Accuracy (%)
**Semi-quantitative**							
**Low-BPE**							
WIS (%/sec)	0.94 [0.89, 0.97]	0.74	73 [64, 80] (93/128)	98 [93, 100] (43/44)	99 [94, 100] (93/94)	55 [44, 66] (43/78)	79 [73, 85] (136/172)
TTP (sec)	0.68 [0.61, 0.75]	49.08	88 [81, 93] (113/128)	39 [26, 53] (17/44)	81 [73, 86] (113/140)	53 [36, 69] (17/32)	76 [69, 81] (130/172)
BAT (sec)	0.69 [0.61, 0.76]	22.32	86 [79, 91] (110/128)	52 [38, 66] (23/44)	84 [77, 89] (110/131)	56 [41, 70] (23/41)	77 [70, 83] (133/172)
PEI	0.79 [0.72, 0.85]	0.48	81 [74, 87] (104/128)	68 [53, 80] (30/44)	88 [81, 93] (104/118)	56 [42, 68] (30/54)	78 [71, 83] (134/172)
iAUC	0.92 [0.86, 0.95]	0.27	79 [71, 85] (101/128)	89 [76, 96] (39/44)	95 [89, 98] (101/106)	59 [47, 70] (39/66)	81 [75, 87] (140/172)
**High-BPE**							
WIS (%/sec)	0.93 [0.88, 0.96]	0.78	76 [69, 82] (123/162)	100 [84, 100] (25/25)	100 [96, 100] (123/123)	39 [28, 51] (25/64)	79 [73, 84] (148/187)
TTP (sec)	0.57 [0.50, 0.64]	62.46	98 [94, 100] (159/162)	16 [6, 35] (4/25)	88 [83, 92] (159/180)	57 [25, 84] (4/7)	87 [82, 91] (163/187)
BAT (sec)	0.61 [0.54, 0.68]	8.94	47 [39, 55] (76/162)	72 [52, 86] (18/25)	92 [83, 96] (76/83)	17 [11, 26] (18/104)	50 [43, 57] (94/187)
PEI	0.79 [0.72, 0.84]	0.60	66 [58, 73] (76/162)	84 [65, 94] (21/25)	96 [91, 99] (107/111)	28 [19, 39] (21/76)	68 [61, 75] (128/187)
iAUC	0.89 [0.84, 0.93]	0.32	70 [63, 77] (114/162)	100 [84, 100] (25/25)	100 [96, 100] (114/114)	34 [24, 46] (25/73)	74 [68, 80] (139/187)
**Quantitative**							
**Low-BPE**							
K^{trans} (min^{-1})	0.84 [0.78, 0.89]	0.29	78 [70, 84] (100/128)	75 [60, 86] (33/44)	90 [83, 95] (100/111)	54 [42, 66] (33/61)	77 [70, 83] (133/172)
k_{ep} (min^{-1})	0.93 [0.88, 0.96]	0.46	98 [94, 100] (126/128)	75 [60, 86] (33/44)	92 [86, 96] (126/137)	94 [80, 99] (33/35)	92 [87, 96] (159/172)
v_e	0.58 [0.50, 0.65]	0.57	71 [63, 78] (91/128)	55 [40, 68] (24/44)	82 [74, 88] (91/111)	39 [28, 52] (24/61)	67 [60, 73] (115/172)
**High-BPE**							
K^{trans} (min^{-1})	0.84 [0.78, 0.89]	0.40	62 [54, 69] (100/162)	100 [84, 100] (25/25)	100 [96, 100] (100/100)	29 [20, 39] (25/87)	67 [60, 73] (125/187)
k_{ep} (min^{-1})	0.91 [0.86, 0.94]	0.56	90 [84, 93] (145/162)	84 [65, 94] (21/25)	97 [93, 99] (145/149)	55 [40, 70] (21/38)	89 [83, 93] (166/187)
v_e	0.68 [0.61, 0.75]	0.49	46 [38, 53] (74/162)	88 [69, 97] (22/25)	96 [89,99] (74/77)	20 [14, 29] (22/110)	51 [44, 58] (96/187)

Unless otherwise specified, data are percentages, followed by 95%CI in brackets and proportions in parentheses. AUCs are expressed as decimal numbers followed by 95%CI in parentheses.

UF-DCE ultrafast dynamic contrast-enhanced, *BPE* background parenchymal enhancement, *WIS* wash-in slope, *TTP* time-to-peak, *BAT* bolus arrival time, *iAUC* initial area under the curve in 60 sec, K^{trans} volume transfer constant, k_{ep} rate constant, v_e extravascular extracellular space volume, *HER2* human epidermal growth factor 2, *TNBC* triple-negative breast cancer, *pCR* pathologic complete response.

Table S9: DeLong test for receiver operator characteristic curve of semi-quantitative and quantitative parameters in the low-BPE subgroup

Parameter	WIS (%/sec)	TTP (sec)	BAT (sec)	PEI	iAUC	K^{trans} (min^{-1})	k_{ep} (min^{-1})	v_e
**Semi-quantitative**								
WIS (%/sec)	/	< 0.001	< 0.001	< 0.001	0.03	0.001	0.64	< 0.001
TTP (sec)	< 0.001	/	0.98	0.15	< 0.001	0.01	< 0.001	0.15
BAT (sec)	< 0.001	0.98	/	0.04	< 0.001	0.01	< 0.001	0.21
PEI	< 0.001	0.15	0.04	/		0.21	0.004	0.01
iAUC	0.03	< 0.001	< 0.001	< 0.001	/	0.02	0.70	< 0.001
**Quantitative**								
K^{trans} (min^{-1})	0.001	0.01	0.01	0.21	0.02	/	0.02	0.00
k_{ep} (min^{-1})	0.64	< 0.001	< 0.001	0.004	0.70	0.02	/	
v_e	< 0.001	0.15	0.21	0.01	< 0.001	0.00	< 0.001	/

Data were p values. $p < 0.05$ indicates significant difference between parameters in the test.
BPE background parenchymal enhancement, *WIS* wash-in slope, *TTP* time-to-peak, *BAT* bolus arrival time, *iAUC* initial area under the curve in 60 sec, K^{trans} volume transfer constant, k_{ep} rate constant, v_e extravascular extracellular space volume.

Table S10: DeLong test for receiver operator characteristic curve of semi-quantitative and quantitative parameters in the high-BPE subgroup

Parameter	WIS (%/sec)	TTP (sec)	BAT (sec)	PEI	iAUC	K^{trans} (min^{-1})	k_{ep} (min^{-1})	v_e
**Semi-quantitative**								
WIS (%/sec)	/	< 0.001	< 0.001	0.001	0.01	< 0.001	0.62	< 0.001
TTP (sec)	< 0.001	/	0.65	0.01	< 0.001	< 0.001	< 0.001	0.10
BAT (sec)	< 0.001	0.65	/	0.02	< 0.001	0.001	< 0.001	0.36
PEI	0.001	0.01	0.02	/	0.01	0.28	0.05	0.21
iAUC	0.01	< 0.001	< 0.001	0.01	/	0.76	0.52	0.43
**Quantitative**								
K^{trans} (min^{-1})	< 0.001	< 0.001	0.001	0.28	0.76	/	0.12	0.03
k_{ep} (min^{-1})	0.62	< 0.001	< 0.001	0.05	0.52	0.12	/	< 0.001
v_e	< 0.001	0.10	0.36	0.21	0.43	0.03	< 0.001	/

Data were p values. $p < 0.05$ indicates significant difference between parameters in the test.
BPE background parenchymal enhancement, *WIS* wash-in slope, *TTP* time-to-peak, *BAT* bolus arrival time, *iAUC* initial area under the curve in 60 sec, K^{trans} volume transfer constant, k_{ep} rate constant, v_e extravascular extracellular space volume.

Table S11: Repeatability and reproducibility of semi-quantitative and quantitative parameters

Parameter	R1 vs R1*	R1 vs R2
Semi-quantitative		
WIS (%/sec)	0.92 (0.85, 0.95)	0.87 (0.78, 0.92)
TTP (sec)	0.94 (0.91, 0.96)	0.78 (0.66, 0.87)
BAT (sec)	0.89 (0.82, 0.93)	0.77 (0.65, 0.86)
PEI	0.92 (0.87, 0.95)	0.81 (0.70, 0.88)
iAUC	0.93 (0.87, 0.97)	0.84 (0.75, 0.90)
Quantitative		
K^{trans} (min^{-1})	0.92 (0.87, 0.95)	0.86 (0.78, 0.92)
k_{ep} (min^{-1})	0.96 (0.93, 0.97)	0.87 (0.77, 0.91)
v_e	0.89 (0.82, 0.93)	0.79 (0.68, 0.87)

Data are expressed as decimal numbers, with 95%CI in parentheses.
WIS wash-in slope, *TTP* time to peak, *BAT* bolus arrival time, *iAUC* initial area under the curve in 60 sec, K^{trans} volume transfer constant, k_{ep} rate constant, v_e extravascular extracellular space volume, *CI* confidence interval.
R1: Reader 1, R1*: the second measurement of R1 after a one-month interval, R2: Reader 2.

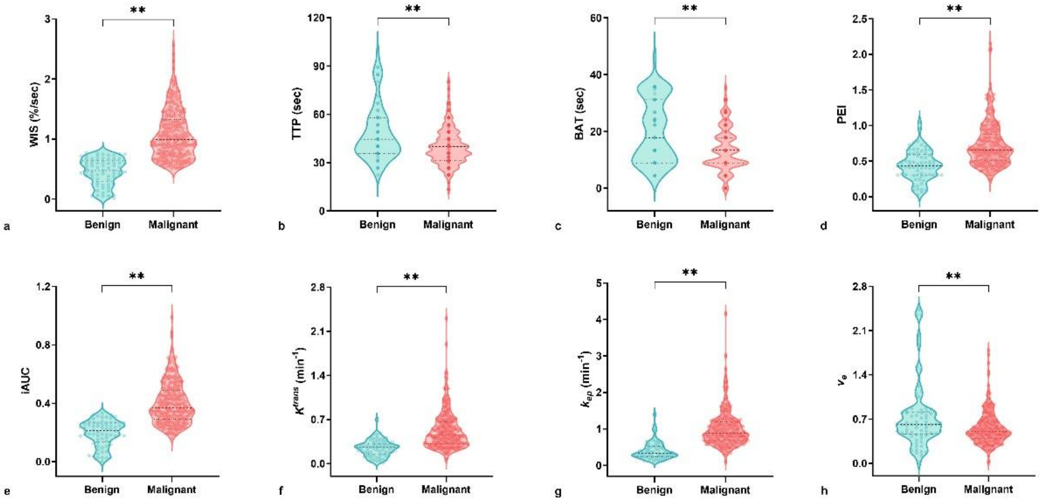

Figure S1: Violin plots of semi-quantitative and quantitative parameters between benign and malignant breast lesions. *WIS* wash-in slope, *TTP* time-to-peak, *BAT* bolus arrival time, *PEI* peak enhancement intensity, *iAUC* initial area under the curve in 60 sec, K^{trans} volume transfer constant, k_{ep} rate constant, v_e extravascular extracellular space volume.

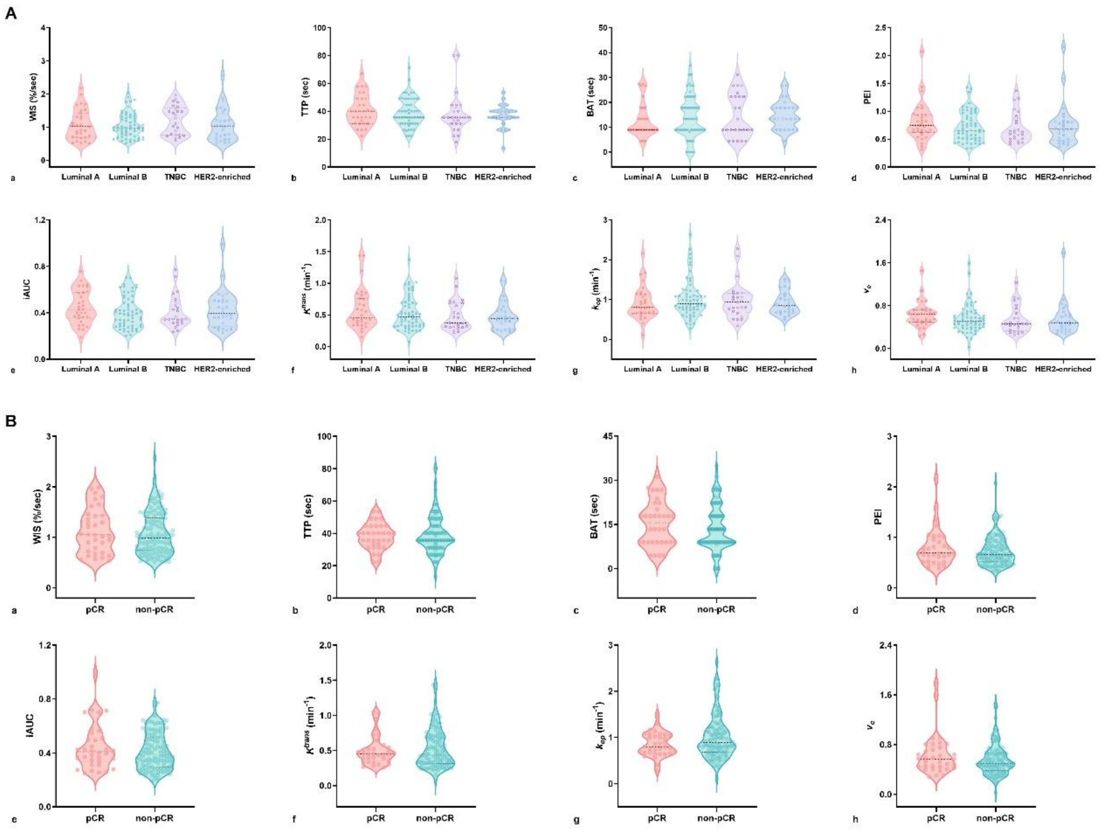

Figure S2: Violin plots of semi-quantitative and quantitative parameters among four molecular subtypes (A) and between pCR and non-pCR groups (B). *WIS* wash-in slope, *TTP* time-to-peak, *BAT* bolus arrival time, *PEI* peak enhancement intensity, *iAUC* initial area under the curve in 60 sec, K^{trans} volume transfer constant, k_{ep} rate constant, v_e extravascular extracellular space volume.
